# ﻿Phylogeny-based reinterpretation of the genus *Blaps* Fabricius, 1775 (Coleoptera, Tenebrionidae, Blaptinae) from China, with description of two new species

**DOI:** 10.3897/zookeys.1244.145585

**Published:** 2025-07-08

**Authors:** Bao-Yue Zhang, Xiu-Min Li

**Affiliations:** 1 Key Laboratory of Zoological Systematics and Application of Hebei Province; College of Life Sciences, Hebei University, Baoding 071002, China Hebei University Baoding China

**Keywords:** Darkling beetles, molecular phylogenetics, morphology, taxonomy

## Abstract

*Blaps* is the most species-rich genus in the subtribe Blaptina (Tenebrionidae, Blaptinae), with 205 described species. In this study, we reconstructed a preliminary phylogenetic tree based on cytochrome c oxidase subunit I sequences from 34 species of *Blaps* and *Nalepa*. Based on both molecular and morphological evidence, we propose a new classification for the genus *Blaps*. Consequently, *Blaps (Blaps) holcus* Ren, 2016 is transferred from the subgenus Blaps to the subgenus Ablapsis and Nalepa is reinstated as a subgenus of Blaps, *Nalepa*, **stat. resurr.** Furthermore, two new species are described and illustrated from China: *Blaps (Blaps) pedinoides* Li, **sp. nov.** and *Blaps (Nalepa) undulata* Li, **sp. nov.** This work provides valuable molecular and morphological data for the phylogenetic study of the subtribe Blaptina.

## ﻿Introduction

The Blaptina Latreille, 1817 is the most numerous subtribe within the tribe Blaptini Leach, 1815 (Kamiński et al. 2024). It comprises 348 species and 11 genera. Its range covers a significant portion of the Palaearctic Region, as well as northern areas of the Afrotropical and Indo-Malayan regions (Chigray and Kirejtshuk 2023). *Blaps* is the most species-rich genus in the subtribe Blaptina, with 205 described species, which are primarily distributed in the Palaearctic region (Nabozhenko and Chigray 2020). Currently, *Blaps* was subdivided into four subgenera: *Ablapsis* Reitter, 1887 (20 spp./ssp.), *Blaps* Fabricius, 1775 (182 spp./ssp.), *Dineria* Motschulsky, 1860 (two spp.), and *Arenoblaps* G.S. Medvedev, 1999 (one sp.). The taxonomy of the genus *Blaps* is complicated and confusing despite some progress made with recent revisions (Soldati et al. 2017; Chigray and Kirejtshuk 2023). Molecular studies also have indicated that *Blaps* is likely paraphyletic (Condamine et al. 2013; Li et al. 2023).

*Nalepa* was erected by Reitter in 1887 as a subgenus of *Blaps* with *Blaps cylindracea* Reitter, 1887 as the type species (type locality: Amdo) (Reitter 1887). Reitter (1893) later revised the status of *Nalepa* and elevated it to a genus. Currently, *Nalepa* comprises seven species, which are distributed in the southern Qinghai, northwestern Sichuan, and eastern Xizang, China (Li et al. 2022). *Nalepa* and *Blaps* are similar in their external morphology and the shape of the aedeagus. However, they can be differentiated by male characteristics: outer spur of protibia much smaller than inner one in *Nalepa* (spurs of protibia slightly different in size in *Blaps*). The monophyly of *Nalepa* and its phylogenetic placement within Blaptini still require verification, particularly with molecular data.

In this study, we reconstructed a preliminary phylogenetic tree to assess the taxonomic status of the genus *Nalepa* in relation to *Blaps*. Additionally, two new species are described and illustrated based on morphological and molecular evidence. This study provides valuable data for understanding phylogenetic relationships within the subtribe Blaptina.

## ﻿Materials and methods

### ﻿Morphological examination

In total, 23 samples of two species were examined for this study, which are deposited at the Museum of Hebei University, Baoding, China (**MHBU**).

The photos were taken with the following imaging system: Canon EOS 5D Mark III (Canon Inc., Tokyo, Japan) connected to a Laowa FF 100 mm F2.8 CA-Dreamer Macro 2× or Laowa FF 25 mm F2.8 Ultra Macro 2.5–10× (Anhui Changgeng Optics Technology Co., Hefei, China). Multiple images were stacked to construct the final figures. Montaged images were edited using Adobe Photoshop v. 22.1.0 to form the final figure plates.

Label data are presented verbatim. A slash (/) separates text on different lines of label.

### ﻿Taxon sampling, DNA extraction, PCR amplification, and sequencing

Specimens were collected in the field from Sichuan, Xizang, and Yunnan China. Detailed information for all the samples used in this study (including those without molecular data) is provided in Suppl. material 1: table S1.

DNA was extracted from leg muscle tissue of adults using the Insect DNA Isolation Kit (BIOMI-GA, Hangzhou, China) following the manufacturer’s protocols. The DNA extracted was stored at −20 °C. Fragment of mitochondrial molecular marker (cytochrome oxidase subunit I, *COI*) was amplified with the primers F2183 and R3014 (Folmer et al. 1994). The profile of the PCR amplification consisted of an initial denaturation step at 94 °C for 4 min, 35 cycles of denaturation at 94 °C for 1 min, annealing for 45 s, an extension at 72 °C for 1 min, and a final 8 min extension step at 72 °C. PCR was performed using TaKaRa Ex Taq (TaKaRa, Dalian, China). PCR products were subsequently checked by 1% agarose gel electrophoresis and sequencing was performed at General Biol Co. (Anhui, China). Detailed information for the samples used in this study is provided in Suppl. material 1: table S1.

### ﻿Phylogenetic analyses

In total, we used 147 sequences including 31 published sequences for the in-group. We used the previously four published sequences of three species of Platyscelidini Lacordaire, 1859 as outgroups, which have been considered as close relatives of the tribe Blaptini (Kamiński et al. 2021).

Phylogenetic analysis was based on the cytochrome coxidase subunit I gene fragment by maximum likelihood (ML). A best-fit model was tested according to the corrected Akaike’s Information Criterion (AIC) using ModelFinder (included in IQ-TREE) with PhyloSuite v. 1.2.2 (Zhang et al. 2020). The ML tree search was performed in IQ-TREE v. 1.6.8 (Nguyen et al. 2015) that was also plugged into PhyloSuite. The ML tree was inferred using an edge-linked partition model for 5000 ultrafast bootstraps (1000 replicates) (Minh et al. 2013). Support for each node is represented by ultrafast bootstrap values (uBV).

## ﻿Results and discussion

**Figure 1.**
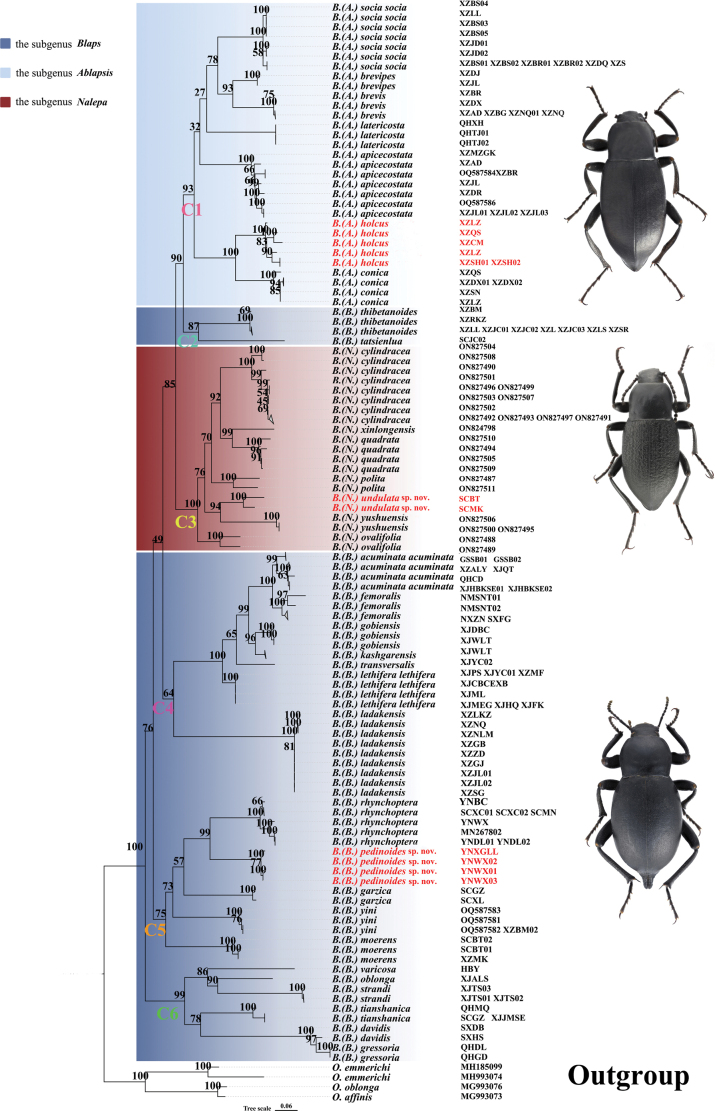
Maximum-likelihood phylogenetic tree based on *COI* sequences (667 bp) within the genus *Blaps*.

**Figure 2.**
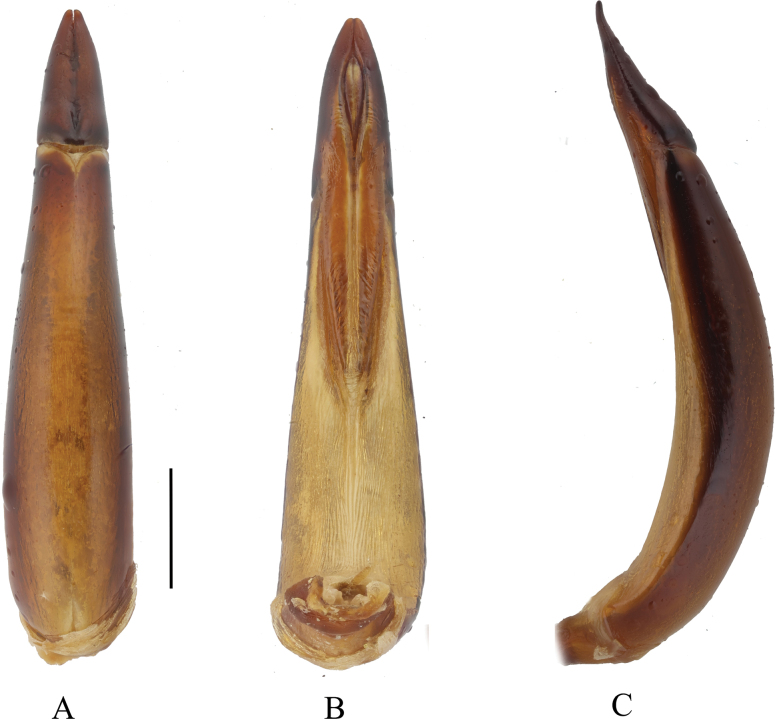
*Blaps (Blaps) holcus* Ren, 2016, aedeagus. **A.** Dorsal view; **B.** Ventral view; **C.** Lateral view. Scale bars: 1.0 mm.

The IQ-TREE analysis yielded a topology based on *COI* sequences (667 bp), and the preliminary phylogenetic relationships were hypothesized from 147 samples (Fig. 1). In-group individuals were classified into six clades: Clade C1, Clade C2, Clade C3, Clade C4, Clade C5, and Clade C6. Six species of the subgenus Ablapsis corresponded to the well-supported Clade C1 (uBV = 93%), with the exception of *Blaps (Blaps) holcus* Ren, 2016, which was originally assigned to the subgenus Blaps. We also re-examined the aedeagal structure of *Blaps (Blaps) holcus* Ren, 2016 (Fig. 2), and found that it aligns with the characteristics of the subgenus Ablapsis (smaller parameres in relation to the basal piece of the aedeagus, parameres slightly curved to dorsal side apically in lateral view). Based on both molecular and morphological evidence, we propose transferring *Blaps (Blaps) holcus* Ren, 2016 to the subgenus Ablapsis. Seven species of the genus *Nalepa*, including the new species, corresponded to the well-supported Clade C3 (uBV = 100%). However, the phylogenetic results did not support Clade C3 as a valid genus; rather, it is more appropriately classified as a subgenus of *Blaps*. *Nalepa* and *Blaps* can be distinguished based on the characteristics of the protibial spurs. However, notable similarities are also observed in the aedeagus of many species (Li et al. 2022; Chigray and Kirejtshuk 2023). Consequently, we propose reinstating *Nalepa* as a subgenus of *Blaps* based on the phylogenetic results: *Nalepa*, stat. resurr. Thus, *Blaps* comprises five subgenera: *Ablapsis* Reitter, 1887 (20 spp./ssp.), *Arenoblaps* G.S. Medvedev, 1999 (one sp.), *Blaps* Fabricius, 1775 (183 spp./ssp.), *Dineria* Motschulsky, 1860 (two spp.), and *Nalepa* Reitter, 1887 (eight spp.).

In this study, the phylogenetic results indicate that species of the subgenus Blaps correspond to multiple clades, and it presents a polyphyletic group. Most species of *Blaps* belong to the subgenus Blaps Fabricius, 1775, which exhibits high species diversity and a wide distribution range; the habitats of these species have also undergone significant changes. The morphological structures and aedeagus structures of species in different habitats display considerable diversity. Research also indicates that *Blaps* is likely paraphyletic based on the molecular data (Condamine et al. 2013; Soldati et al. 2017; Li et al. 2023). In the taxonomic history of the tribe Blaptini, where numerous species were originally described in the genus *Blaps*, including species that are now assigned to genera belonging to other subtribes (Löbl et al. 2008). However, due to the complex taxonomic history, morphological diversity, and other factors, establishing a clear classification system inter-subgenus and inter-species within *Blaps* remains a significant challenge. Future efforts should focus on accumulating extensive molecular datasets, morphological characteristics, and a denser sampling for the genus *Blaps* to verify the taxonomic status of subgenera and species.

### ﻿Taxonomic descriptions

#### ﻿ Blaps (Blaps) pedinoides

Taxon classificationAnimaliaColeopteraTenebrionidae

Li sp. nov.

0040FC1C-5C79-5093-95DF-CC37D183F842

https://zoobank.org/549CBAA6-114C-4BF5-A10A-CB599743FF43

##### Type material.

***Holotype***: China • ♂ (MHBU HBU(E)339974); Sichuan, Jiulong County/ 28°37.89'N, 99°39.16'E/ Alt 1917m/ 2. VIII. 2020/ Ming-min Ma leg. ***Paratypes***: China • 1♀ [in ethanol] (MHBU HBU(E)339975), same data as holotype; China • 1♂ [1♂in ethanol] (MHBU HBU(E)339976); Yunan; Weixi County, Tacheng Township/ 27°36.51'N, 99°23.87'E/ Alt 1987m/ 11.VIII.2021/ Zhong-hua Wei leg; China •1♂ [in ethanol] (MHBU HBU(E)339977); Yunan; Weixi County, Tacheng Township/ 27°26.19'N, 99°20.98'E/ 13.VII. 2021/ Alt 2409m/ Guo-dong Ren leg; China •1♂ [in ethanol] (MHBU HBU(E)339978); Yunan, Shangri-la County, Nixi Township/ 28°01.41'N, 99°28.02'E/ Alt 2778m/7.VIII.2015/ Guo-dong Ren leg; China •1♂ [in ethanol] (MHBU HBU(E)339979); Yunan, Weixi County, Jingzhong Township/ 27°38.09'N, 99°21.78'E/ Alt 2635m/ 13.VII. 2021/ Zhong-hua Wei leg.

##### Description.

**Figure 3.**
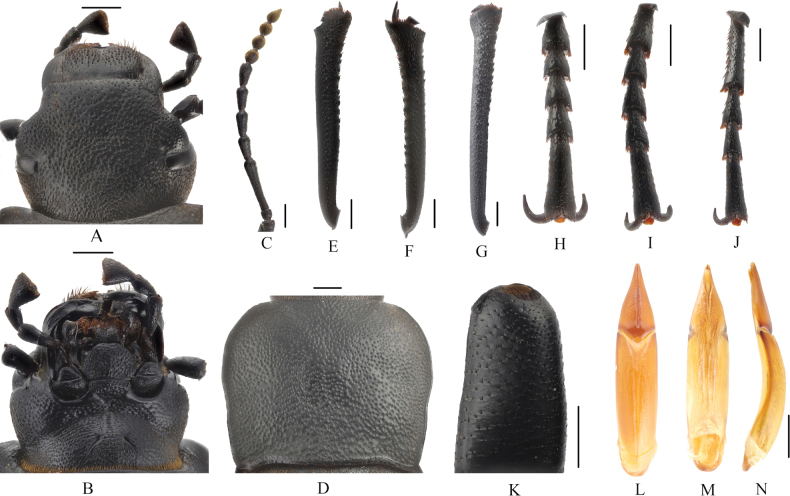
*Blaps (Blaps) pedinoides* Li, sp. nov. **A.** Head, dorsal view; **B.** Head, ventral view; **C.** Antenna; **D.** Pronotum; **E.** Protibial; **F.** Mesotibia; **G.** Metatibia; **H.** Protarsus; **I.** Mesotarsus; **J.** Metatarsus; **K.** Profemur apically; **L–N.** Aedeagus; **L.** Dorsal view; **M.** Ventral view; **N.** Lateral view. Scale bars: 1.0 mm.

**Figure 4.**
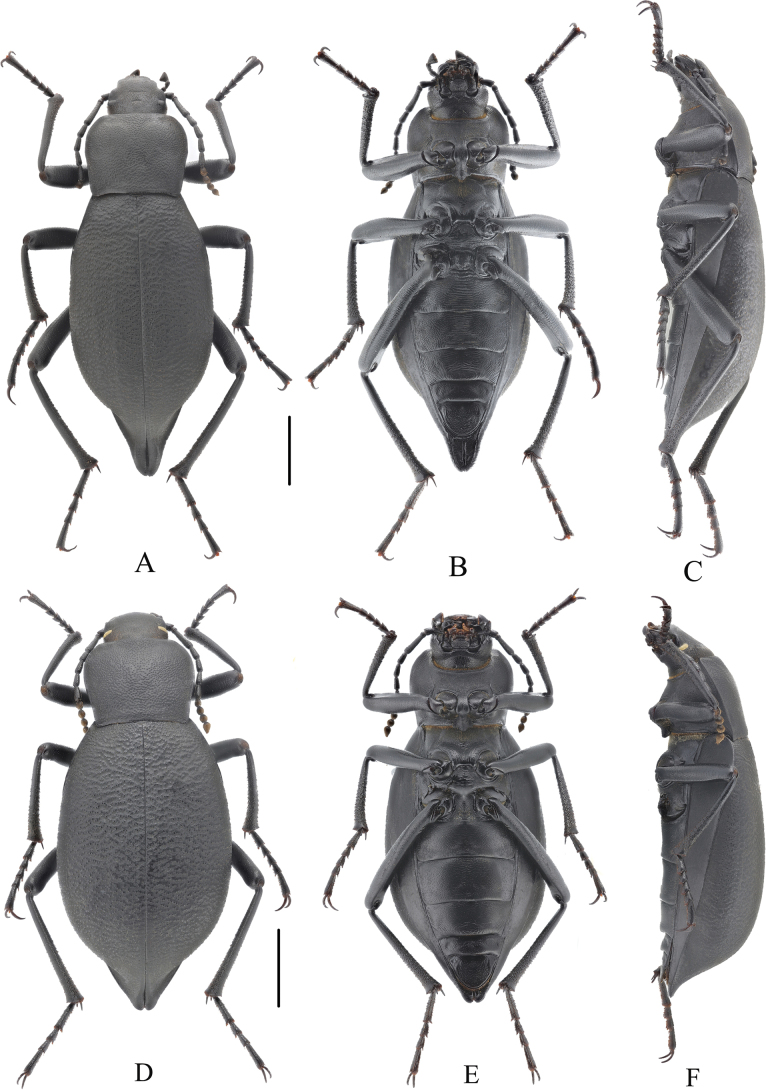
Habitus of *Blaps (Blaps) pedinoides* Li, sp. nov. **A–C.** Male, holotype; **A.** Dorsal view; **B.** Ventral view; **C.** Lateral view; **D–F.** Female, paratype; **D.** Dorsal view; **E.** Ventral view; **F.** Lateral view. Scale bars: 5 mm.

**Male** (Figs 3, 4A–C). Body elongated, length 28.5–29.0 mm, width 9.5–10.0 mm; dull black, rough, oval-oblong, opaque.

***Head*** (Fig. 3A, B). Anterior margin of epistome straight. Head slightly convex, with dense, coarse punctation. Mentum oval; base of mentum straight. Antennae long, antennomere XI reaching base of pronotum when directed backwards, antennomeres III–VII cylindrical, VIII–X spherical, XI spindle (Fig. 3C). Ratio of length/width of antennomeres II–XI 6.6(8.2): 37.8(9.0): 16.6(9.0): 17.8(8.7): 17.3(9.0): 15.6(9.0): 10.0(10.1): 10.0(9.9): 10.4(9.8): 11.7(9.7).

***Prothorax*** (Fig. 3D). Base of pronotum as wide as the base of elytra. Pronotum slightly transverse, 1.3 times as wide as long, 1.8 times as wide as head, widest before the middle, lateral margins from widest portion toward anterior angles arcuated, then straightly converging towards posterior margin; anterior angles rounded-obtuse; posterior angles weakly obtuse; lateral edges entirely margined, ratio of width at the anterior margin to the widest part and base 38: 63: 53. Disc rough, surface with dense, coarse punctations with large punctures. Prosternal process nearly triangular; middle of posterior margin weakly extended backward, with transverse wrinkles.

***Pterothorax*.** Elytra elongate, ovoid, 1.7 times as long as wide, long arcuated; widest at the middle, 3.0 times as long and 1.4 times as wide as pronotum, 2.5 times as wide as head. Lateral elytral border not entirely visible dorsally. Scutellum small. Disc flat, elytral surface rough, with dense granules, and granules converge into wrinkles. Apex of elytra slowly sloping, with small granules. Elytral mucro obvious, 2.3 mm long. Pseudopleuron smooth. Abdomen shiny, without hair tuft/short setae between 1^st^ and 2^nd^ abdominal ventrites, ventrite 1^st^ with flat large tubercle in middle, 1^st^–3^rd^ ventrites with transverse wrinkles medially and irregular rugosity, granules on both sides, abdominal ventrites 4^th^–5^th^ with sparse punctures.

***Legs*** (Fig. 3E–K). Legs long. Inner side of pro- and mesotibiae slightly curved, metatibiae straight, expanded apically. Ratio of length of pro-, meso- and metatibiae 13: 13: 17; ratio of length (width) of metatarsomeres I–IV 19.4(5.5): 10.3(5.2): 10.0(5.0): 16.8(4.3).

***Aedeagus*.** (Fig. 3L–N). Length 4.8 mm and width 1.0 mm. Parameres length 1.6 mm and width 0.75 mm, with conical shape; parameres widest at the base, apex sharp, lateral margins arcuately converging from base to 1/3, then straightly converging from basal 1/3 to apex in dorsal view, parameres straight line up to the apex in lateral view.

**Female** (Fig. 4D–F). Body length 24.0 mm, width 11.0 mm. Body wider and more robust than in male. Elytral mucro shorter, 1.0 mm long. Head as wide as outerocular distance, pronotum 1.4 times as wide as long, elytra 1.5 times as long as wide. Antennae nearly as long as male, antennomeres X reaching base of pronotum when directed backwards, antennomeres III–VII cylindrical, VIII–X spherical, XI spindle.

##### Etymology.

The name derives from ancient Greek πεδινός (pedinos), flat, referring to the flattened elytra of the new species

##### Distribution.

China: Sichuan, Yunnan.

##### Diagnosis.

The new species is morphologically very similar to B. (B.) rhynchoptera Fairmaire, 1886, and it is also sister with B. (B.) rhynchoptera in the phylogeny tree, but it can be distinguished from the latter by the following male characters: (1) without hair tuft/short setae between 1^st^ and 2^nd^ abdominal ventrites (with hair tuft/short setae between 1^st^ and 2^nd^ abdominal ventrites in B. (B.) rhynchoptera); (2) lateral margin of pronotum weakly broading from base to 2/3, then toward anterior angles arcuated, widest before middle (pronotum widest at middle in B. (B.) rhynchoptera).

Additionally, the new species is morphologically similar to B. (B.) japonensis Marseul, 1879 but can be distinguished from the latter by the following male characters: (1) without hair tuft/short setae between 1^st^ and 2^nd^ abdominal ventrites (with hair tuft/short setae between 1^st^ and 2^nd^ abdominal ventrites in B. (B.) japonensis); (2) lateral margin of pronotum broading from base to 2/3, then toward anterior angles arcuated, widest before the middle (lateral margin of pronotum arcuated, widest at middle in B. (B.) japonensis); (3) parameres conical, apex sharp (parameres bottleneck-shaped, apex sharp in B. (B.) japonensis).

#### ﻿ Blaps (Nalepa) undulata

Taxon classificationAnimaliaColeopteraTenebrionidae

Li sp. nov.

A5BDC709-9C42-58BA-87B5-E639B4A21855

https://zoobank.org/23AB13AF-6356-4A75-82E1-B927E4B7A6E8

##### Type specimens.

***Holotype***: China •♂ (MHBU HBU(E)339980); Sichuan, Batang County; 30°12.45'N, 99°15.59'E/ Alt. 3055 m/ 15.VII.2021/ Xiu-min Li leg. ***Paratypes***: China •10♂♂6♀♀ [2♂♂ in ethanol] (MHBU HBU(E)339981-339996); same data as holotype; China • 1♀ [in ethanol]; Xizang, Markam County, Zangxoi Township/ 29°74.01'N, 98°44.52'E/ 21.VII.2020/ Alt. 3050 m/ Ming-min Ma leg.

##### Description.

**Figure 5.**
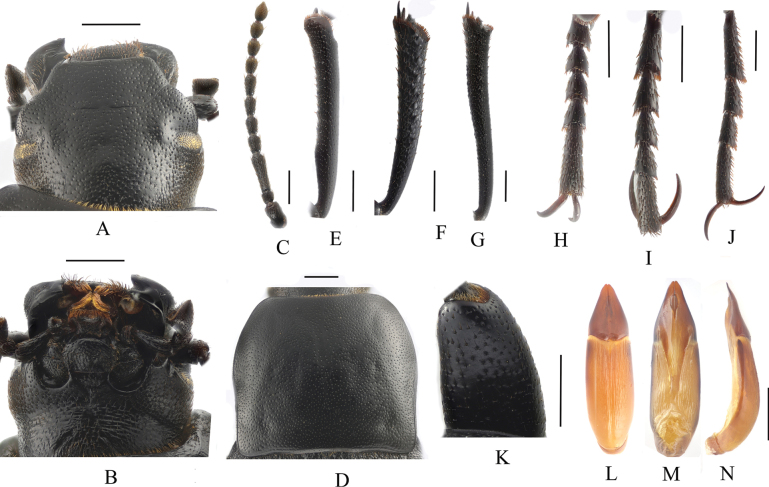
*Blaps (Nalepa) undulata* Li, sp. nov. **A.** Head, dorsal view; **B.** Head, ventral view; **C.** Antenna; **D.** Pronotum; **E.** Protibia; **F.** Mesotibia; **G.** Metatibia; **H.** Protarsus; **I.** Mesotarsus; **J.** Metatarsus; **K.** Profemur apically; **L–N.** Aedeagus; **L.** Dorsal view; **M.** Ventral view; **N.** Lateral view. Scale bars: 1.0 mm.

**Figure 6.**
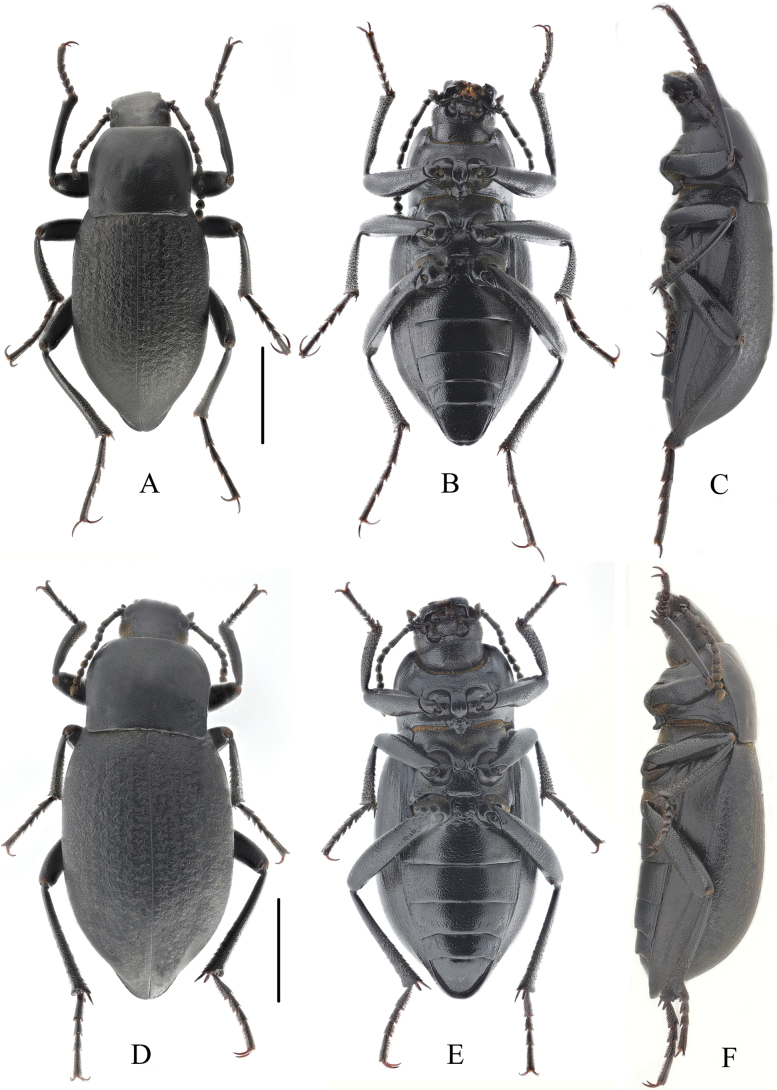
Habitus of *Blaps (Nalepa) undulata* Li, sp. nov. **A–C.** Male, holotype; **A.** Dorsal view; **B.** Ventral view; **C.** Lateral view; **D–F.** Female, paratype; **D.** Dorsal view; **E.** Ventral view; **F.** Lateral view. Scale bars: 5 mm.

**Male** (Figs 5, 6A–C). Body length 17.0–19.0 mm, width 6.5–7.0 mm; black, opaque.

***Head*** (Fig. 5A, B). Anterior margin of epistome straight. Head slightly convex, with sparse, fine punctation. Mentum oval, base of mentum straight. Antennae long, antennomere XI reaching base of pronotum when directed backwards, antennomeres III–VII cylindrical, VIII–X long spherical, XI spindle (Fig. 5C). Ratio of length/width of antennomeres II–XI 10.0(10.6): 36.3(12.5): 16.5(11.1): 16.1(10.3): 17.2(10.0): 19.0(9.5): 14.3(10.1): 14.2(12.0): 14.6(13.0): 19.0(11.0).

***Prothorax*** (Fig. 5D). Pronotum slightly transverse, 1.2 times as wide as long, 1.7 times as wide as head. Widest at the middle, lateral margins slightly emarginated from base to middle, then toward anterior angles arcuated; lateral edges entirely margined; ratio of width at the anterior margin to the widest part and base 26: 45: 43. Disc smooth and convex, surface with dense, fine, small punctation. With two rounds depressions before posterior angles. Apex of prosternal process obtuse, obliquely sloping behind procoxae, distinctly projecting beyond the margin of prosternum.

***Pterothorax*.** Elytra ovoid, 1.6 times as long as wide, weakly arcuated, widest at the middle, 2.5 times as long as and 1.3 times as wide as pronotum, 2.2 times as wide as head. Lateral elytral border not entirely visible dorsally. Scutellum hiding. Disc flattened in middle, rough, with undulate wrinkles. Apex of elytra slowly sloping, obtuse. Without elytral mucro. Abdominal ventrites 1^st^–3^rd^ with irregular fine wrinkles, abdominal ventrites 4^th^–5^th^ with dense punctations.

***Legs*** (Fig. 5E–K). Legs long. Inner side of pro- and metatibiae slightly curved, mesotibiae straight, expanded apically. Ratio of length of pro-, meso- and metatibiae 9.0: 9.0: 12.0, and ratio of length (width) of metatarsomeres I–IV 19.4(5.5): 10.3(5.2): 10.0(5.0): 16.8(4.3).

***Aedeagus*** (Fig. 5L–N). Length 3.3 mm and width 1.0 mm. Parameres length 1.1 mm and width 0.9 mm, conical; parameres wide and convex at base, apex obtuse, lateral margin arcuately narrowing from base to 1/3, then straightly narrowing from basal 1/3 to apex in dorsal view; slightly curved, narrowed almost in a straight line up to the apex in lateral view.

**Female** (Fig. 6D–F). Body length 18.0–19.5 mm, width 8.0–9.0 mm. Body wider and more robust than in male. Elytral without mucro. Head as wide as outterocular distance. Pronotum 1.3 times as wide as long, elytra 1.6 times as long as wide. Antennae shorter than in male, reaching posterior 2/3 of pronotum when directed backwards, antennomeres III–VII cylindrical, VIII–X elongate spherical, XI spindle. Pro- and metatibiae straight.

##### Etymology.

This species is named from the Latin adjective “undulata”, meaning wavy, in reference to the wavy wrinkles on the elytra.

##### Distribution.

China, Sichuan, Xizang.

##### Diagnosis.

The new species is a sister to *Blaps (Nalepa) yushuensis* Li & Ren, 2022 in the phylogenetic analysis. However, it is easy to distinguished from *Blaps (Nalepa) yushuensis* Li & Ren, 2022 and other species of the subgenus Nalepa by the following characters: (1) parameres conical, apex rounded (parameres apex acuminate in other species); (2) elytra rough, with undulate wrinkles (elytral surface smooth, with sparse punctures and shallow wrinkles in other species).

## Supplementary Material

XML Treatment for Blaps (Blaps) pedinoides

XML Treatment for Blaps (Nalepa) undulata

## ﻿Additional information

### Use of AI

No use of AI was reported.

### Author ORCIDs

Bao-Yue Zhang https://orcid.org/0009-0008-9251-4792

Xiu-Min Li https://orcid.org/0000-0003-0575-1869

### Data availability

All of the data that support the findings of this study are available in the main text or Supplementary Information.

## Supplementary materials

Supplementary material 1 List of specimens used in this study Data type docx Explanation note List of specimens used in this study with the corresponding accession number. https://binary.pensoft.net/file/1369676This dataset is made available under the Open Database License (http://opendatacommons.org/licenses/odbl/1.0/). The Open Database License (ODbL) is a license agreement intended to allow users to freely share, modify, and use this Dataset while maintaining this same freedom for others, provided that the original source and author(s) are credited.Bao-Yue Zhang, Xiu-Min Li
