# Supplementary material for: ﻿Phylogeny-based reinterpretation of the genus Blaps Fabricius, 1775 (Coleoptera, Tenebrionidae, Blaptinae) from China, with description of two new species
Source: Zookeys. 2025 Jul 8;1244:99–111. doi: 10.3897/zookeys.1244.145585 (PMC12264499; doi:10.3897/zookeys.1244.145585)
Supplement: Supplementary material 1 — List of specimens used in this study [file zookeys-1244-099_article-145585__-s001.docx]

**Table S1.** List of specimens used in this study with the corresponding accession number.

|  | ID | Species / Subspecies | Sampling locality | Elevation (m) | Date of collection | Collector(s) | Preservation | Accession numbers（COI） |
| --- | --- | --- | --- | --- | --- | --- | --- | --- |
| 1 | XJHBKSE01 | *Blaps* (*Blaps*) *acuminata acuminata* | Baiyanghe, Hoboksar, Xinjiang, China | 890 | 11.Ⅴ.2024 | X. Li *et al*. | Ethanol |  |
| 2 | XJHBKSE02 | *Blaps* (*Blaps*) *acuminata acuminata* | Baiyanghe, Hoboksar, Xinjiang, China | 890 | 11.Ⅴ.2024 | X. Li *et al*. | Ethanol |  |
| 3 | XJALT | *Blaps* (*Blaps*) *acuminata acuminata* | Qiemuerqieke,Altay, Xinjiang, China | 766 | 14.Ⅴ.2024 | X. Li *et al*. | Ethanol |  |
| 4 | XJQT | *Blaps* (*Blaps*) *acuminata acuminata* | Xibeiwan, Qitai, Xinjiang, China | 762 | 31.Ⅶ.2018 | X. Bai *et al*. | Ethanol |  |
| 5 | QHCD | *Blaps* (*Blaps*) *acuminata acuminata* | Zhenqin, Chindu, Qinghai, China | n/a | 19.Ⅵ.2018 | L. Wang *et al*. | Ethanol |  |
| 6 | GSSB01 | *Blaps* (*Blaps*) *acuminata acuminata* | Dangchengwan, Subei, Gansu, China | 2803 | 13.Ⅶ.2019 | X. Bai *et al*. | Ethanol |  |
| 7 | GSSB02 | *Blaps* (*Blaps*) *acuminata acuminata* | Dangchengwan, Subei, Gansu, China | 2702 | 13.Ⅶ.2019 | X. Bai *et al*. | Ethanol |  |
| 8 | NMSNT01 | *Blaps* (*Blaps*) *femoralis* | Sonid Zuoqi, Neimenggu, China | n/a | 3.Ⅷ.2017 | *G. Ren et al.* | Ethanol |  |
| 9 | NMSNT02 | *Blaps* (*Blaps*) *femoralis* | Sonid Youqi, Neimenggu, China | n/a | 2.Ⅷ.2017 | G. Ren *et al.* | Ethanol |  |
| 10 | NXZN | *Blaps* (*Blaps*) *femoralis* | Zhongning, Ningxia, China | n/a | 18.Ⅳ.2017 | G. Ren *et al.* | Ethanol |  |
| 11 | SXFG | *Blaps* (*Blaps*) *femoralis* | Fugu, Shaanxi, China | n/a | 25.Ⅶ.2018 | G. Ren *et al.* | Ethanol |  |
| 12 | XJPS | *Blaps* (*Blaps*) *kashgarensis* | Kangkir, Pishan, Xinjiang, China | 2323 | 6.VIII.2018 | X. Bai *et al*. | Ethanol |  |
| 13 | XJYC01 | *Blaps* (*Blaps*) *kashgarensis* | Kokyar, Yecheng, Xinjiang, China | 2280 | 7.VIII.2018 | X. Bai *et al*. | Ethanol |  |
| 14 | XJMF | *Blaps* (*Blaps*) *kashgarensis* | Niya, Minfeng, Xinjiang, China | 1428 | 5.VIII.2018 | X. Bai *et al*. | Ethanol |  |
| 15 | XJYC02 | *Blaps* (*Blaps*) *kashgarensis* | Yecheng, Xinjiang, China | 2622 | 7.VIII.2018 | X. Bai *et al*. | Ethanol |  |
| 16 | XJWLT01 | *Blaps* (*Blaps*) *gobiensis* | Urad Qianqi, Neimenggu, China | 1023 | 25.VII.2018 | X. Bai *et al*. | Ethanol |  |
| 17 | XJWLT02 | *Blaps* (*Blaps*) *gobiensis* | Urad Qianqi, Neimenggu, China | 1023 | 25.VII.2018 | X. Bai *et al*. | Ethanol |  |
| 18 | XJDBC | *Blaps* (*Blaps*) *gobiensis* | Dabancheng, XinJiang, China | 1069 | 11.Ⅴ.2024 | X. Li *et al*. | Ethanol |  |
| 19 | XJCBCEXB | *Blaps* (*Blaps*) *transversalis* | Qapqal Xibe, Xinjiang, China | 1472 | 7.Ⅴ.2024 | X. Li *et al*. | Ethanol |  |
| 20 | XJMEG | *Blaps* (*Blaps*) *lethifera lethifera* | Miaorgou, Urumqi, XinJiang, China | 1423 | 21.Ⅴ.2024 | X. Li *et al*. | Ethanol |  |
| 21 | XJHQ | *Blaps* (*Blaps*) *lethifera lethifera* | Hongqi, Jimsar, Xinjiang, China | 782 | 31.VII.2018 | X. Bai *et al*. | Ethanol |  |
| 22 | XJFK | *Blaps* (*Blaps*) *lethifera lethifera* | Fukang, Xinjiang, China | 626 | 1.Ⅷ.2018 | X. Bai *et al*. | Ethanol |  |
| 23 | XJML | *Blaps* (*Blaps*) *lethifera lethifera* | Baiyanghe, Mulei, Xinjiang, China | 1353 | 29.VII.2018 | X. Bai *et al*. | Ethanol |  |
| 24 | XZGB | *Blaps* (*Blaps*) *ladakensis* | Lungzhong, Gamba, Xizang, China | 4405 | 20.VIII.2019 | X. Li *et al*. | Ethanol |  |
| 25 | XZZD | *Blaps* (*Blaps*) *ladakensis* | Qangzê, Zada, Xizang, China | 4822 | 25.VIII.2015 | X. Bai *et al*. | Ethanol |  |
| 26 | XZGJ | *Blaps* (*Blaps*) *ladakensis* | Wumbudangsang, Gê’gyai, Xizang, China | 4422 | 23.VIII.2015 | X. Bai *et al*. | Ethanol |  |
| 27 | XZJL01 | *Blaps* (*Blaps*) *ladakensis* | Zongga, Gyirong, Xizang, China | 4480 | 26.Ⅵ.2018 | X. Bai *et al*. | Ethanol |  |
| 28 | XZJL02 | *Blaps* (*Blaps*) *ladakensis* | Zheba, Gyirong, Xizang, China | n/a | 1.VIII.2014 | G. Ren *et al*. | Ethanol |  |
| 29 | XZSG | *Blaps* (*Blaps*) *ladakensis* | Sakya, Xizang, China | 4507 | 13.VIII.2019 | X. Li *et al*. | Ethanol |  |
| 30 | XZNQ | *Blaps* (*Blaps*) *ladakensis* | Namarqê, Nagqu, Xizang, China | 4604 | 3.VIII.2019 | G. Ren *et al*. | Ethanol |  |
| 31 | XZNLM | *Blaps* (*Blaps*) *ladakensis* | Nyalam, Xizang, China | 4348 | 6.VIII.2023 | X. Bai *et al*. | Ethanol |  |
| 32 | XZLKZ | *Blaps* (*Blaps*) *ladakensis* | Garkry mount, Longkatz, Xizang, China | 4515 | 2.VIII.2019 | X. Li *et al*. | Ethanol |  |
| 33 | XJTS01 | *Blaps* (*Blaps*) *strandi* | Tianshan, Xinjiang, China | 2222 | 29.VII.2018 | X. Bai *et al*. | Ethanol |  |
| 34 | XJTS02 | *Blaps* (*Blaps*) *strandi* | Tianshan, Xinjiang, China | 2222 | 29.VII.2018 | X. Bai *et al*. | Ethanol |  |
| 35 | XJTS03 | *Blaps* (*Blaps*) *strandi* | Tianshan, Xinjiang, China | 2222 | 29.VII.2018 | X. Bai *et al*. | Ethanol |  |
| 36 | XJALS | *Blaps* (*Blaps*) *oblonga* | Bayan Hot, Alxa Zuoqi, Xinjiang, China | 1157 | 5.VI.2024 | G. Ren *et al*. | Ethanol |  |
| 37 | HBY | *Blaps* (*Blaps*) *varicosa* | Yu, Hebei, China | n/a | n/a | G. Ren *et al*. | Ethanol |  |
| 38 | QHDL | *Blaps* (*Blaps*) *gressoria* | Hoit Taria, Delingha, Qinghai, China | 3315 | 30.VIII.2015 | X. Bai *et al.* | Ethanol |  |
| 39 | QHGD | *Blaps* (*Blaps*) *gressoria* | Guide, Qinghai, China | 2335 | 14.VIII.2019 | X. Bai *et al.* | Ethanol |  |
| 40 | SXHS | *Blaps* (*Blaps*) *davidis* | Hengshan, Qinghai, China | n/a | 23.Ⅳ.2017 | G. Ren *et al*. | Ethanol |  |
| 41 | SXDB | *Blaps* (*Blaps*) *davidis* | Dingbian, Qinghai, China | n/a | 23.Ⅳ.2017 | G. Ren *et al*. | Ethanol |  |
| 42 | SCGZ | *Blaps* (*Blaps*) *tianshanica* | Tuoba, Garzê, Sichuan, China | 3347 | 15.VI.2028 | L. Wang *et al.* | Ethanol |  |
| 43 | XJJMSE | *Blaps* (*Blaps*) *tianshanica* | Xindi, Jimsar, Xinjiang, China | 1425 | 29.VII.2018 | X. Bai *et al*. | Ethanol |  |
| 44 | QHMQ | *Blaps* (*Blaps*) *tianshanica* | Dawu, Maqên, Qinghai, China | 3828 | 18.VI.2028 | L. Wang *et al.* | Ethanol |  |
| 45 | SCXC01 | *Blaps* (*Blaps*) *rhynchoptera* | Maan, Xichang, Sichuan, China | 1703 | 2.VIII.2015 | X. Bai *et al*. | Ethanol |  |
| 46 | SCXC02 | *Blaps* (*Blaps*) *rhynchoptera* | Maan, Xichang, Sichuan, China | 1703 | 2.VIII.2015 | X. Bai *et al*. | Ethanol |  |
| 47 | SCMN | *Blaps* (*Blaps*) *rhynchoptera* | Manshuiwan, Mianning, Sichuan, China | 1744 | 1.VIII.2020 | M. Ma *et al*. | Ethanol |  |
| 48 | YNDL01 | *Blaps* (*Blaps*) *rhynchoptera* | Yinqiao, Dali, Yunnan, China | 1974 | 12.VII.2017 | X. Bai *et al*. | Ethanol |  |
| 49 | YNDL02 | *Blaps* (*Blaps*) *rhynchoptera* | Xizhou, Dali, Yunnan, China | 2008 | 12.VII.2017 | G. Ren *et al*. | Ethanol |  |
| 50 | YNWX | *Blaps* (*Blaps*) *rhynchoptera* | Baohe, Weixi, Yunnan, China | 2230 | 13.VII.2021 | G. Ren *et al*. | Ethanol |  |
| 51 | n/a | *Blaps* (*Blaps*) *rhynchoptera* | n/a | n/a | n/a | M. Zhao *et al.* | Ethanol | MN267802 |
| 52 | YNBC | *Blaps* (*Blaps*) *rhynchoptera* | Jizu Shan, Binchuan, Yunnan, China | 2240 | 12.VII.2017 | G. Ren *et al*. | Ethanol |  |
| 53 | YNWX01 | *Blaps* (*Blaps*) *pedinoides* **sp. nov.** | Tacheng, Weixi, Yunnan, China | 1987 | 11.VII.2017 | Z. Wei *et al.* | Ethanol |  |
| 54 | YNWX02 | *Blaps* (*Blaps*) *pedinoides* **sp. nov.** | Tacheng, Weixi, Yunnan, China | 2409 | 13.VII.2017 | G. Ren *et al*. | Ethanol |  |
| 55 | YNWX03 | *Blaps* (*Blaps*) *pedinoides* **sp. nov.** | Jingzhong, Weixi, Yunnan, China | 2635 | 13.VII.2017 | Z. Wei *et al.* | Ethanol |  |
| 56 | YNXGLL | *Blaps* (*Blaps*) *pedinoides* **sp. nov.** | Nixi, Shangri-la, Yunnan, China | 2778 | 7.VIII.2015 | G. Ren *et al*. | Ethanol |  |
| 57 | SCGZ | *Blaps* (*Blaps*) *garzica* | Batang, Garzê, Sichuan, China | 3055 | 15.VII.2021 | X. Li *et al*. | Ethanol |  |
| 58 | SCXL | *Blaps* (*Blaps*) *garzica* | Marê, Xinlong, Sichuan, China | 4028 | 14.VIII.2016 | X. Li *et al*. | Ethanol |  |
| 59 | SCBT01 | *Blaps* (*Blaps*) *moerens* | Dangba, Batang, Sichuan, China | 2647 | 13.VIII.2016 | X. Li *et al*. | Ethanol |  |
| 60 | XZMK | *Blaps* (*Blaps*) *moerens* | Rongmê, Markom, Xizang, China | 2650 | 12.VIII.2016 | X. Li *et al*. | Ethanol |  |
| 61 | SCBT02 | *Blaps* (*Blaps*) *moerens* | Chubalung, Batang, Sichuan, China | 2530 | 26.VII.2022 | G. Ren *et al.* | Ethanol |  |
| 62 | XZBM01 | *Blaps* (*Blaps*) *yini* | Tangmai, Bomi, Xizang, China | n/a | 17.VIII.2020 | Z. Hu *et al.* | Ethanol | OQ587581 |
| 63 | XZBM02 | *Blaps* (*Blaps*) *yini* | Yi’ong, Bomi, Xizang, China | 2082 | 30.VII.2021 | G. Ren *et al.* | Ethanol | OQ587582 |
| 64 | XZBM03 | *Blaps* (*Blaps*) *yini* | Paggai, Bomi, Xizang, China | n/a | 1.VIII.2021 | G. Ren *et al.* | Ethanol | OQ587583 |
| 65 | XZBM02 | *Blaps* (*Blaps*) *yini* | Yi’ong, Bomi, Xizang, China | 2082 | 30.VII.2021 | G. Ren *et al.* | Ethanol |  |
| 66 | XZLL | *Blaps* (*Blaps*) *thibetanoides* | Lhorong, Xizang, China | 4773 | 28.VII.2019 | X. Li *et al.* | Ethanol |  |
| 67 | XZJC01 | *Blaps* (*Blaps*) *thibetanoides* | Gyaca, Xizang, China | 3285 | 28.VII.2019 | X. Li *et al.* | Ethanol |  |
| 68 | XZJC02 | *Blaps* (*Blaps*) *thibetanoides* | Gyaca, Xizang, China | 3285 | 28.VII.2019 | X. Li *et al.* | Ethanol |  |
| 69 | XZL | *Blaps* (*Blaps*) *thibetanoides* | Tunggar, Lang, Xizang, China | n/a | 11.VIII.2014 | G. Ren *et al.* | Ethanol |  |
| 70 | XZZD | *Blaps* (*Blaps*) *thibetanoides* | Zêtang, Xizang, China | 3538 | 13.VIII.2018 | H. Chen *et al.* | Ethanol |  |
| 71 | XZJC03 | *Blaps* (*Blaps*) *thibetanoides* | Lingda, Gyaca, Xizang, China | n/a | 10.VIII.2014 | G. Ren *et al.* | Ethanol |  |
| 72 | XZLS | *Blaps* (*Blaps*) *thibetanoides* | Lhasa, Xizang, China | 3627 | 13.VIII.2015 | X. Bai *et al.* | Ethanol |  |
| 73 | XZSR | *Blaps* (*Blaps*) *thibetanoides* | Baidui, Sangri, Xizang, China | 3350 | 23.VII.2022 | G. Ren *et al.* | Ethanol |  |
| 74 | XZBM | *Blaps* (*Blaps*) *thibetanoides* | Laka, Bomê, Xizang, China | 2427 | 31.VII.2021 | G. Ren *et al.* | Ethanol |  |
| 75 | XZRKZ | *Blaps* (*Blaps*) *thibetanoides* | Xigazê, Xizang, China | n/a | 26.VII.2014 | G. Ren *et al.* | Ethanol |  |
| 76 | SCJC02 | *Blaps* (*Blaps*) *tatsienlua* | Jinchuan, Sichuan, China | 2582 | 19.VII.2021 | X. Li *et al*. | Ethanol |  |
| 77 | XZSH01 | *Blaps* (*Ablapsis*) *holcus* | Dagzê Co, Xizang, China | 4570 | 16.VIII.2018 | X. Bai *et al.* | Ethanol |  |
| 78 | XZSH02 | *Blaps* (*Ablapsis*) *holcus* | Dagzê Co, Xizang, China | 4570 | 16.VIII.2018 | X. Bai *et al.* | Ethanol |  |
| 79 | XZLZ | *Blaps* (*Ablapsis*) *holcus* | Charong, Lhundup, Xizang, China | 4564 | 8.VIII.2019 | X. Li *et al.* | Ethanol |  |
| 80 | XZLZ | *Blaps* (*Ablapsis*) *holcus* | Loca, Xizang, China | 4015 | 3.VIII.2019 | X. Li *et al.* | Ethanol |  |
| 81 | XZCM | *Blaps* (*Ablapsis*) *holcus* | Comai, Xizang, China | n/a | 8.VIII.2014 | G. Ren *et al.* | Ethanol |  |
| 82 | XZQS | *Blaps* (*Ablapsis*) *holcus* | Qusum , Xizang, China | 4232 | 29.VII.2019 | X. Li *et al.* | Ethanol |  |
| 83 | XZDX01 | *Blaps* (*Ablapsis*) *conica* | Doiling, Damxung, Xizang, China | 4379 | 9.VIII.2019 | X. Li *et al*. | Ethanol |  |
| 84 | XZDX02 | *Blaps* (*Ablapsis*) *conica* | Yangbajain, Damxung, Xizang, China | n/a | 23.VII.2014 | G. Ren *et al.* | Ethanol |  |
| 85 | XZSN | *Blaps* (*Ablapsis*) *conica* | Lhünzê, Xizang, China | 4248 | 30.VII.2019 | X. Li *et al*. | Ethanol |  |
| 86 | XZLZ | *Blaps* (*Ablapsis*) *conica* | Lhünzê, Xizang, China | 4248 | 30.VII.2019 | X. Li *et al*. | Ethanol |  |
| 87 | XZQS | *Blaps* (*Ablapsis*) *conica* | Siu, Qusum, Xizang, China | n/a | 9.VIII.2014 | G. Ren *et al.* | Ethanol |  |
| 88 | XZAD | *Blaps* (*Ablapsis*) *brevis* | Bangmêr, Amdo, Xizang, China | 4763 | 19.VIII.2018 | X. Bai *et al.* | Ethanol |  |
| 89 | XZNQ01 | *Blaps* (*Ablapsis*) *brevis* | Nagqu, Xizang, China | n/a | 15.VIII.2015 | G. Ren *et al.* | Ethanol |  |
| 90 | XZBG | *Blaps* (*Ablapsis*) *brevis* | Bangoin, Xizang, China | 4635 | 3.VIII.2019 | X. Bai *et al.* | Ethanol |  |
| 91 | XZNQ02 | *Blaps* (*Ablapsis*) *brevis* | Nagqu, Xizang, China | 4626 | 10.VIII.2019 | X. Bai *et al.* | Ethanol |  |
| 92 | XZBR | *Blaps* (*Ablapsis*) *brevis* | Xiaqu, Biru, Xizang, China | 4300 | 2.VIII.2019 | X. Bai *et al.* | Ethanol |  |
| 93 | XZDX | *Blaps* (*Ablapsis*) *brevis* | Nyingzhong, Damxung, Xizang, China | 4200 | 4.VIII.2019 | X. Bai *et al*. | Ethanol |  |
| 94 | XZDJ | *Blaps* (*Ablapsis*) *brevipes* | Dinggyê, Xizang, China | n/a | 2.VIII.2014 | G. Ren *et al.* | Ethanol |  |
| 95 | XZJL | *Blaps* (*Ablapsis*) *brevipes* | Zongga, Gyirong, Xizang, China | 4284 | 26.Ⅵ.2018 | X. Bai *et al.* | Ethanol |  |
| 96 | XZBS01 | *Blaps* (*Ablapsis*) *socia socia* | Guoqing, Baxoi, Xizang, China | 4451 | 3.VIII.2021 | G. Ren *et al.* | Ethanol |  |
| 97 | XZBR01 | *Blaps* (*Ablapsis*) *socia socia* | Caqu, Biru, Xizang, China | 4071 | 6.VII.2022 | G. Ren *et al.* | Ethanol |  |
| 98 | XZBS02 | *Blaps* (*Ablapsis*) *socia socia* | Guoqing, Baxoi, Xizang, China | 4451 | 3.VIII.2021 | G. Ren *et al.* | Ethanol |  |
| 99 | XZBR02 | *Blaps* (*Ablapsis*) *socia socia* | Zhala, Biru, Xizang, China | 4211 | 6.VII.2022 | G. Ren *et al.* | Ethanol |  |
| 100 | XZDQ | *Blaps* (*Ablapsis*) *socia socia* | Shagong, Dênqên, Xizang, China | 3711 | 22.VIII.2018 | X. Bai *et al.* | Ethanol |  |
| 101 | XZS | *Blaps* (*Ablapsis*) *socia socia* | Sêrcham, Sog, Xizang, China | 4115 | 21.VIII.2018 | X. Bai *et al.* | Ethanol |  |
| 102 | XZBS03 | *Blaps* (*Ablapsis*) *socia socia* | Baxoi, Xizang, China | 4542 | 23.VII.2021 | G. Ren *et al.* | Ethanol |  |
| 103 | XZBS04 | *Blaps* (*Ablapsis*) *socia socia* | Baxoi, Xizang, China | 3913 | 20.VIII.2018 | X. Bai *et al.* | Ethanol |  |
| 104 | XZLL | *Blaps* (*Ablapsis*) *socia socia* | Nagjog, Lhorong, Xizang, China | 3910 | 30.VII.2019 | G. Ren *et al.* | Ethanol |  |
| 105 | XZBS05 | *Blaps* (*Ablapsis*) *socia socia* | Yiqing, Baxoi, Xizang, China | 4333 | 10.VIII.2016 | X. Li *et al.* | Ethanol |  |
| 106 | XZJD01 | *Blaps* (*Ablapsis*) *socia socia* | Jomda, Xizang, China | 4164 | 8.VIII.2016 | X. Li *et al.* | Ethanol |  |
| 107 | XZJD02 | *Blaps* (*Ablapsis*) *socia socia* | Jomda, Xizang, China | 4043 | 8.VIII.2016 | X. Bai *et al.* | Ethanol |  |
| 108 | QHTJ01 | *Blaps* (*Ablapsis*) *latericosta* | Tianjun, Qinghai, China | 3632 | 6.VIII.2021 | X. Bai *et al.* | Ethanol |  |
| 109 | QHTJ02 | *Blaps* (*Ablapsis*) *latericosta* | Tianjun, Qinghai, China | 3632 | 6.VIII.2021 | X. Bai *et al.* | Ethanol |  |
| 110 | QHXH | *Blaps* (*Ablapsis*) *latericosta* | Wenquan, Xinghai, Qinghai, China | 4000 | 13.VIII.2019 | X. Bai *et al.* | Ethanol |  |
| 111 | XZJL03 | *Blaps* (*Ablapsis*) *apicecostata* | Gyirong, Xizang, China | n/a | 1.VIII.2014 | G. Ren *et al.* | Ethanol |  |
| 112 | XZJL02 | *Blaps* (*Ablapsis*) *apicecostata* | Zongga, Gyirong, Xizang, China | 3746 | 27.Ⅵ.2018 | X. Bai *et al.* | Ethanol |  |
| 113 | XZJL01 | *Blaps* (*Ablapsis*) *apicecostata* | Zongga, Gyirong, Xizang, China | 4051 | 14.VII.2022 | G. Ren *et al.* | Ethanol |  |
| 114 | XZDR01 | *Blaps* (*Ablapsis*) *apicecostata* | Rongxar, Tingri, Xizang, China | n/a | 27.VII.2014 | G. Ren *et al.* | Ethanol | OQ587584 |
| 115 | XZJL | *Blaps* (*Ablapsis*) *apicecostata* | Arza, Ihari, Xizang, China | 4048 | 9.VIII.2019 | G. Ren *et al.* | Ethanol |  |
| 116 | XZDR02 | *Blaps* (*Ablapsis*) *apicecostata* | Rongxar, Dingri, Xizang, China | n/a | 26.VII.2014 | G. Ren *et al.* | Ethanol |  |
| 117 | XZBR | *Blaps* (*Ablapsis*) *apicecostata* | Paingar, Biru, Xizang, China | 5005 | 12.VIII.2015 | X. Bai *et al.* | Ethanol |  |
| 118 | XZBR01 | *Blaps* (*Ablapsis*) *apicecostata* | Xiaqu, Biru, Xizang, China | 4309 | 13.VIII.2015 | X. Bai *et al.* | Ethanol | OQ587586 |
| 119 | XZAD | *Blaps* (*Ablapsis*) *apicecostata* | Amdo, Xizang, China | 4678 | 18.VIII.2018 | X. Bai *et al.* | Ethanol |  |
| 120 | XZMZGK | *Blaps* (*Ablapsis*) *apicecostata* | Maqu, Maizhokunggar, Xizang, China | 3782 | 26.VII.2019 | X. Li *et al.* | Ethanol |  |
| 121 | XZJD | *Blaps* (*Nalepa*) *ovalifolia* | Gyamda, Jomda, Xizang, China | 3630 | 8.VIII.2016 | X. Li *et al.* | Ethanol | ON827488 |
| 122 | SCLH | *Blaps* (*Nalepa*) *ovalifolia* | Xindu, Luhuo, Sichuan, China | 3194 | 6.VIII.2016 | X. Li *et al.* | Ethanol | ON827489 |
| 123 | XZGJ | *Blaps* (*Nalepa*) *cylindracea* | Gyanbê, Gonjo, Xizang, China | 3758 | 8.VIII.2016 | X. Li *et al.* | Ethanol | ON827492 |
| 124 | XZCD01 | *Blaps* (*Nalepa*) *cylindracea* | Lhatog, Chamdo, Xizang, China | 3724 | 9.VIII.2016 | X. Li *et al.* | Ethanol | ON827493 |
| 125 | XZCD02 | *Blaps* (*Nalepa*) *cylindracea* | Toba, Chamdo, Xizang, China | 4017 | 9.VIII.2016 | X. Li *et al.* | Ethanol | ON827497 |
| 126 | XZJD01 | *Blaps* (*Nalepa*) *cylindracea* | Qu’nyido, Jomda,  Xizang, China | 4050 | 8.VIII.2016 | X. Li *et al.* | Ethanol | ON827491 |
| 127 | QHNQ | *Blaps* (*Nalepa*) *cylindracea* | Oyala pass, Nangqên, Qinghai, China | 4432 | 27.VII.2019 | X. Bai *et al.* | Ethanol | ON827502 |
| 128 | XZBQ01 | *Blaps* (*Nalepa*) *cylindracea* | Ya’ngan, Baqen, Xizang, China | 4135 | 21.VIII.2018 | X. Bai *et al.* | Ethanol | ON827503 |
| 129 | XZBQ02 | *Blaps* (*Nalepa*) *cylindracea* | Ya’ngan, Baqen, Xizang, China | 4135 | 21.VIII.2018 | X. Bai *et al.* | Ethanol | ON827507 |
| 130 | QHZD01 | *Blaps* (*Nalepa*) *cylindracea* | Saiqu, Zadoi, Qinghai, China | 4045 | 22.VII.2012 | G. Ren *et al.* | Ethanol | ON827496 |
| 131 | QHZD02 | *Blaps* (*Nalepa*) *cylindracea* | Gesang, Zadoi, Qinghai, China | 4043 | 25.VII.2019 | X. Bai *et al.* | Ethanol | ON827499 |
| 132 | XZLWQ | *Blaps* (*Nalepa*) *cylindracea* | Gyidoi, Riwoqê, Xizang, China | 3837 | 27.VII.2019 | X. Bai *et al.* | Ethanol | ON827501 |
| 133 | XZCD02 | *Blaps* (*Nalepa*) *cylindracea* | Qu’nyido, Jomda, Xizang, China | 4050 | 8.VIII.2016 | X. Li *et al.* | Ethanol | ON827490 |
| 134 | XZMK | *Blaps* (*Nalepa*) *cylindracea* | G214 road, Mangkang, Xizang, China | 2689 | 13.VII.2021 | X. Li *et al.* | Ethanol | ON827504 |
| 135 | XZMK | *Blaps* (*Nalepa*) *cylindracea* | Zom La shan, Mangkang, Xizang, China | 4186 | 12.VIII.2016 | X. Li *et al.* | Ethanol | ON827508 |
| 136 | SCXL01 | *Blaps* (*Nalepa*) *quadrata* | Le’an, Xinlong, Sichuan, China | 4015 | 14.VIII.2016 | X. Li *et al.* | Ethanol | ON827494 |
| 137 | SCLH01 | *Blaps* (*Nalepa*) *quadrata* | Simu, Luhuo, Sichuan, China | 3180 | 3.VIII.2016 | X. Li *et al.* | Ethanol | ON827509 |
| 138 | SCLH02 | *Blaps* (*Nalepa*) *quadrata* | G350 road, Luhuo, Sichuan, China | 3094 | 17.VII.2021 | X. Li *et al.* | Ethanol | ON827505 |
| 139 | SCLH03 | *Blaps* (*Nalepa*) *quadrata* | Simu, Luhuo, Sichuan, China | 3180 | 3.VIII.2016 | X. Li e*t al*. | Ethanol | ON827510 |
| 140 | SCXL | *Blaps* (*Nalepa*) *xinlongensis* | Mari, Xinlong, Sichuan, China | 4028 | 14.VIII.2016 | X. Li *et al.* | Ethanol | ON827498 |
| 141 | QHYS01 | *Blaps* (*Nalepa*) *yushuensis* | Jyêgu, Yushu, Qinghai, China | 4008 | 24.VII.2019 | X. Bai *et al.* | Ethanol | ON827500 |
| 142 | QHYS02 | *Blaps* (*Nalepa*) *yushuensis* | Shanglaxiu, Yushu, Qinghai, China | 4227 | 21.VII.2012 | G. Ren *et al.* | Ethanol | ON827495 |
| 143 | XZCD | *Blaps* (*Nalepa*) *yushuensis* | Chaiwei, Qamdo, Xizang, China | 3408 | 23.VIII.2018 | X. Bai *et al.* | Ethanol | ON827506 |
| 144 | SCBT | *Blaps* (*Nalepa*) *undulata* **sp. nov*.*** | Batang, Sichuan, Xizang, China | 3055 | 15.VII.2021 | X. Li *et al.* | Ethanol |  |
| 145 | SCMK | *Blaps* (*Nalepa*) *undulata* **sp. nov.** | Zangxoi, Markam, Xizang, China | 3050 | 21.VII.2020 | M. Ma *et al.* | Ethanol |  |
| 146 | SCLH | *Blaps* (*Nalepa*) *polita* | Rinda, Luhuo, Sichuan, China | 3092 | 3.VIII.2016 | X. Li *et al.* | Ethanol | ON827487 |
| 147 | SCGZ | *Blaps* (*Nalepa*) *polita* | Yagra, Garzê, Sichuan, China | 36670 | 5.VIII.2016 | X. Li *et al.* | Ethanol | ON827511 |
| 148 |  | *Oodescelis emmerichi* | Niubei liang, Zhashui, Shaanxi, China | n/a | 22.VII.2011 | X. Zhu *et al.* | Ethanol | MH185099 |
| 149 |  | *Oodescelis affinis* | Daban, Tekes, Xinjiang, China | n/a | 29.Ⅴ.2006 | Y. Ba *et al.* | Ethanol | MG993073 |
| 150 |  | *Oodescelis emmerichi* | Huang shian, Xixia, Henan, China | n/a | 18.VIII.2008 | X. Zhu *et al.* | Ethanol | MG993074 |
| 151 |  | *Oodescelis oblonga* | Kurdnin, Tokkuztara, Xinjiang, China | n/a | 4.VIII.2007 | C. Zhang *et al* | Ethanol | MG993076 |
